# Precise cloning and tandem integration of large polyketide biosynthetic gene cluster using *Streptomyces* artificial chromosome system

**DOI:** 10.1186/s12934-015-0325-2

**Published:** 2015-09-16

**Authors:** Hee-Ju Nah, Min-Woo Woo, Si-Sun Choi, Eung-Soo Kim

**Affiliations:** Department of Biological Engineering, Inha University, Incheon, 402-751 Korea

**Keywords:** *Streptomyces* artificial chromosome, Pathway tandem integration, Polyketide biosynthetic gene cluster

## Abstract

**Background:**

Direct cloning combined with heterologous expression of a secondary metabolite biosynthetic gene cluster has become a useful strategy for production improvement and pathway modification of potentially valuable natural products present at minute quantities in original isolates of actinomycetes. However, precise cloning and efficient overexpression of an entire biosynthetic gene cluster remains challenging due to the ineffectiveness of current genetic systems in manipulating large-sized gene clusters for heterologous as well as homologous expression.

**Results:**

A versatile *Escherichia coli*-*Streptomyces* shuttle bacterial artificial chromosomal (BAC) conjugation vector, pSBAC, was used along with a cluster tandem integration approach to carry out homologous and heterologous overexpression of a large 80-kb polyketide biosynthetic pathway gene cluster of tautomycetin (TMC), which is a protein phosphatase PP1/PP2A inhibitor and T cell-specific immunosuppressant. Unique *Xba*I restriction sites were precisely inserted at both border regions of the TMC biosynthetic gene cluster within the chromosome of TMC-producing *Streptomyces* sp. CK4412, followed by site-specific recombination of pSBAC into the flanking region of the TMC gene cluster. The entire TMC gene cluster was then rescued as a single giant recombinant pSBAC by *Xba*I digestion of the chromosomal DNA as well as subsequent self-ligation. Next, the recombinant pSBAC construct containing the entire TMC cluster in *E. coli* was directly conjugated into model *Streptomyces* strains, resulting in rapid and enhanced TMC production. Moreover, introduction of the TMC cluster-containing pSBAC into wild-type *Streptomyces* sp. CK4412 as well as a recombinant *S. coelicolor* strain resulted in a chromosomal tandem repeat of the entire TMC cluster with 14-fold and 5.4-fold enhanced TMC productivities, respectively.

**Conclusions:**

The 80-kb TMC biosynthetic gene cluster was isolated in a single integration vector, pSBAC. Introduction of TMC biosynthetic gene cluster in TMC non-producing strains has resulted in similar amount of TMC production yield. Moreover, over-expression of TMC biosynthetic gene cluster in original producing strain and recombinant *S. coelicolor* dramatically increased TMC production. Thus, this strategy can be employed to develop a custom overexpression scheme of entire metabolite pathway clusters present in actinomycetes.

**Electronic supplementary material:**

The online version of this article (doi:10.1186/s12934-015-0325-2) contains supplementary material, which is available to authorized users.

## Background

Microbial natural products are a major resource for drug discovery and development programs mainly due to their superior structural diversity and complexity [1]. Isolation and characterization of the natural products of biosynthetic gene clusters have accelerated our understanding of the molecular mechanisms driving natural product biosynthesis and even guided the rational redesign of natural products through biosynthetic gene manipulation [2]. Since most microbial biosynthetic genes are clustered within chromosomes, identification of the entire biosynthetic gene cluster is relatively straightforward. Unfortunately, some of these biosynthetic genes are derived from non-culturable or not amenable to genetic manipulation microorganisms and thus do not easily express the target compounds [3]. To bypass such intrinsic limitations and achieve functional expression of uncharacterized potentially-valuable natural product biosynthetic pathways, a relatively well-characterized heterologous host should be utilized [4, 5]. Recent genome mining approaches have also identified a significant number of metabolite biosynthetic gene clusters, some of which must be expressed in a heterologous host. Synthetic biology techniques have also made it possible to produce novel and/or improved natural products through reconstitution of biosynthetic gene clusters in an appropriate host system [3, 6–8].

Since the gene clusters responsible for the biosynthesis of microbial secondary metabolites are typically as large as 100 kb, appropriate vector systems capable of cloning an entire gene cluster as well as transferring these genetic segments between different hosts are necessary. Capturing an entire biosynthetic gene cluster in a single *E. coli* clone can facilitate the genetic manipulation of secondary metabolite biosynthetic pathways using the PCR-targeted gene replacement method [9–11]. Recently, a new *E*. *coli*-*Streptomyces* shuttle bacterial artificial chromosomal (BAC) vector system, pSBAC, which conveniently switches single-copy to high-copy replication in *E. coli* as well as utilizes the phage ΦBT1 *attP*-*int* site-specific integration system, was successfully used for the heterologous expression of a meridamycin (*mer*) biosynthesis gene cluster in *S. lividans* [12]. Specifically, to clone and express the entire *mer* cluster, a total genomic DNA library was first constructed through ligation of *Eco*RI-digested pSBAC and *Mfe*I-digested total genomic DNA, followed by intergeneric conjugation of the *mer* cluster containing pSBAC into *S. lividans* [12]. In this case, *Mfe*I sites were present just outside of the *mer* cluster, and the large DNA fragment digested in the CHEF gel was also directly ligated with the *Mfe*I-compatible *Eco*RI-digested pSBAC [12]. Therefore, it is more desirable to develop a general cloning strategy to quickly capture an entire biosynthetic gene cassette without depending on endogenous restriction sites in the cluster or a large DNA fragment ligation process.

The plasmid rescue method is a technique for cloning and identifying the region of a locus adjacent to where an exogenous DNA fragment is inserted in the chromosome [13–15]. Based on the capability of pSBAC to accommodate large DNA fragments, the recombinant pSBAC rescue method was carried out to precisely clone a large (approximately 80 kb) polyketide biosynthetic gene cluster for tautomycetin (TMC), which is a protein phosphatase PP1/PP2A inhibitor and T-cell-specific immunosuppressant [16–18]. Unique *Xba*I restriction sites were first inserted at both border regions of the TMC biosynthetic gene cluster within the chromosome of TMC-producing *Streptomyces* sp. CK4412, followed by site-specific recombination of pSBAC into the flanking region of the TMC gene cluster. Moreover, introduction of the TMC cluster-containing pSBAC into wild-type *Streptomyces* sp. CK4412 as well as a recombinant *S. coelicolor* strain resulted in a chromosomal tandem repeat of the entire TMC cluster with 40-fold enhanced TMC productivities. This strategy consisting of site-specific restriction site insertion, recombinant pSBAC plasmid rescue, intergeneric conjugation, and cluster tandem repeat introduction can be employed to develop a custom overexpression scheme of entire metabolite pathway clusters present in actinomycetes (Additional file 1: Table S1).

## Results

### PCR-targeting of unique restriction enzyme sites into borders of TMC gene cluster

**Fig. 1:**
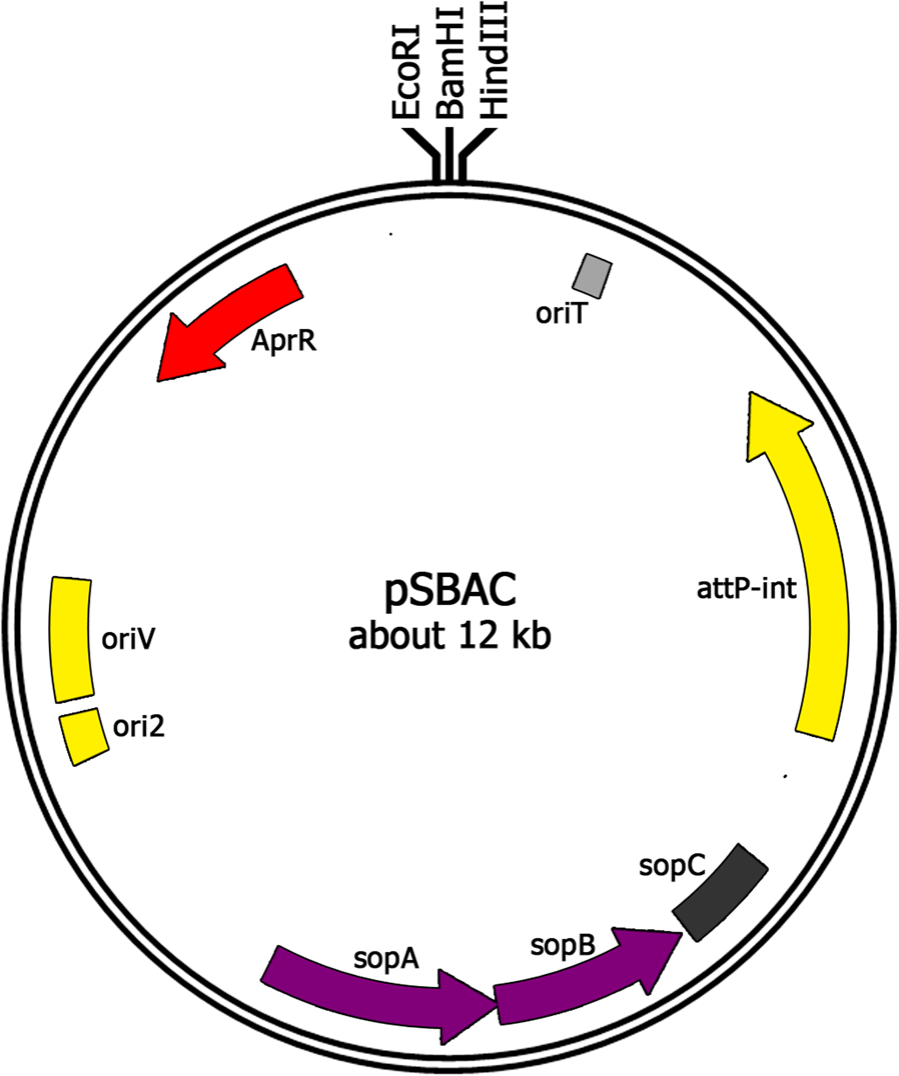
Map of pSBAC. Essential components of the vector are indicated. *Ori2* and *oriV*, replication origins; SopA-C, partitioning system; *aacIII(IV)*, apramycin resistance gene; *oriT*, origin of transfer; ΦBT1 *attP*-*int*, integration system; Unique restriction enzyme recognition sites, *Bam*HI, *Hind*III, and *Eco*RI

**Fig. 2:**
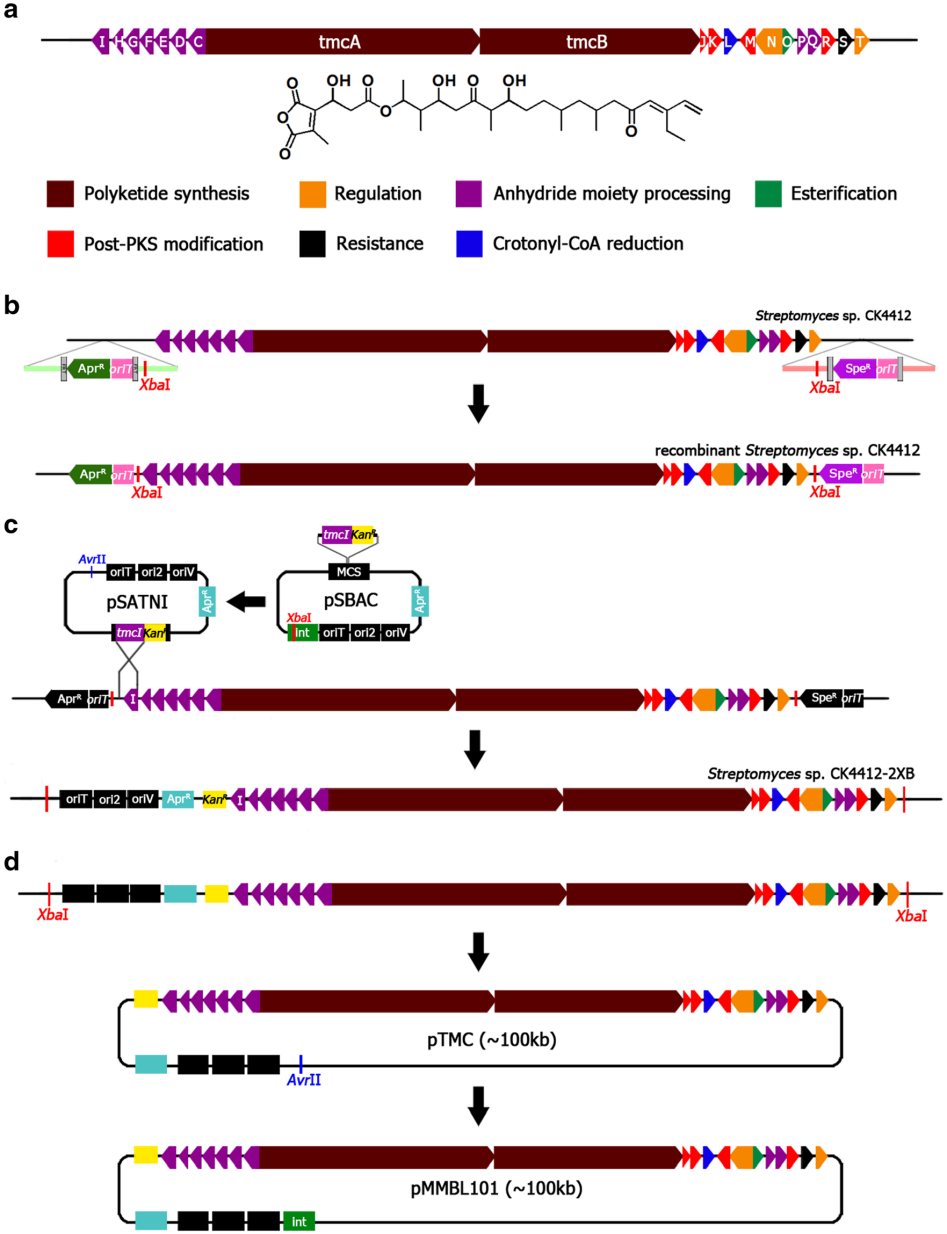
Schematic description of pMMBL101 construction. *TmcA* and *tmcB*, type I polyketide synthase; *tmcC*-*I* and *tmcP*-*Q*, diakylmaleic anhydride moiety processing; *tmcJ* and *tmcK*, decarboxylase; *tmcL*, crotonyl-CoA reductase; *tmcM*, dehydratase; *tmcN* and *tmcT*, pathway-specific regulator; *tmcO*, thioesterase; *tmcR*, cytochrome P450; *tmcS*, transporter. **a** Tautomycetin structure and its biosynthetic gene cluster organization in *Streptomyces* sp. CK4412. **b** Insertion of *Xba*I recognition sequences into both flanking regions of *tmc* cluster via PCR-targeting system. **c** Modification of pSBAC and introduction of modified pSBAC (pSATNI) into *Streptomyces* sp. CK4412 chromosome. **d** *Xba*I digestion of CK4412 chromosome and self-ligation of digested chromosomal DNA to generate pTMC. After construction of pTMC, *attP*-*int* gene was inserted into *Avr*II recognition site of pTMC to generate pMMBL101

An *E. coli*-*Streptomyces* BAC conjugation vector, pSBAC (Fig. 1), has been successfully applied for heterologous expression of the entire meridamycin (*mer*) biosynthetic gene cluster [12]. The entire *mer* gene cluster (~95 kb) could be captured in a single pSBAC clone by straightforward restriction enzyme digestion due to the presence of unique restriction enzyme *Mfe*I sites in border regions of the *mer* biosynthetic gene cluster. In contrast, most secondary metabolite biosynthetic gene clusters such as the TMC gene cluster do not possess unique restriction sites in border regions (Fig. 2a). To apply the pSBAC cloning system to metabolite gene clusters lacking unique restriction enzyme sites in their border regions, we inserted unique *Xba*I restriction enzyme sites into border regions of the TMC biosynthetic gene cluster in the *Streptomyces* sp. CK4412 chromosome using PCR-targeted gene insertion. For this, two DNA fragments, each containing a selection marker, *oriT*, and *Xba*I resctiction enzyme site, were synthesized and precisely inserted into TMC border-containing cosmids, pTMC2982 and pTMC2290, in *E. coli*. The modified cosmids were then conjugated into *Streptomyces* CK4412, followed by target sequence-specific recombination at the borders of the TMC gene cluster (Fig. 2b). The resulting ex-conjugants were isolated based on the selection markers and confirmed to possess the correct *Xba*I insertions by PCR analysis and sequencing (Additional file 2: Fig. S1).

### Precise cloning of entire TMC biosynthetic gene cluster as a single giant recombinant pSBAC

The typical cloning method for large-sized DNA fragment isolation requires extra care so as to avoid unintended DNA fragmentation. Alternatively, the in vivo plasmid rescue method can be used to isolate a particular chromosomal locus through recovery of adjacent DNA sequences [13–15]. Here, we applied the plasmid rescue technique using pSBAC in order to clone a large DNA fragment containing the TMC biosynthetic gene cluster. A 3480-bp *tmcI* DNA fragment containing the gene at the left end of the cluster was first cloned into *attP*-*int* deleted pSBAC plasmid (named pSATNI), followed by conjugation into *Streptomyces* sp. CK4412. The presence of the *tmcI* fragment allowed pSATNI vector to integrate into the left site of the TMC biosynthetic gene cluster as a result of targeted homologous recombination (Fig. 2c). Genomic DNA from kanamycin-resistant conjugants was isolated and digested by *Xba*I restriction enzyme. *Xba*I-digested total chromosomal DNA fractions were self-ligated, followed by transformation into *E*. *coli* cells. DNA was then isolated from the transformants and analyzed by PCR, restriction enzyme digestion, and sequencing. Analysis revealed that the entire TMC biosynthetic gene cluster was successfully cloned as a single recombinant pSBAC vector (Additional file 3: Fig. S2). Finally, the DNA fragment containing ФBT1 *attP*-*int* was re-introduced into the rescued recombinant pSBAC vector and named pMMBL101 (Fig. 2d).

### Heterologous expression of TMC biosynthetic gene cluster in *Streptomyces* strains

**Fig. 3:**
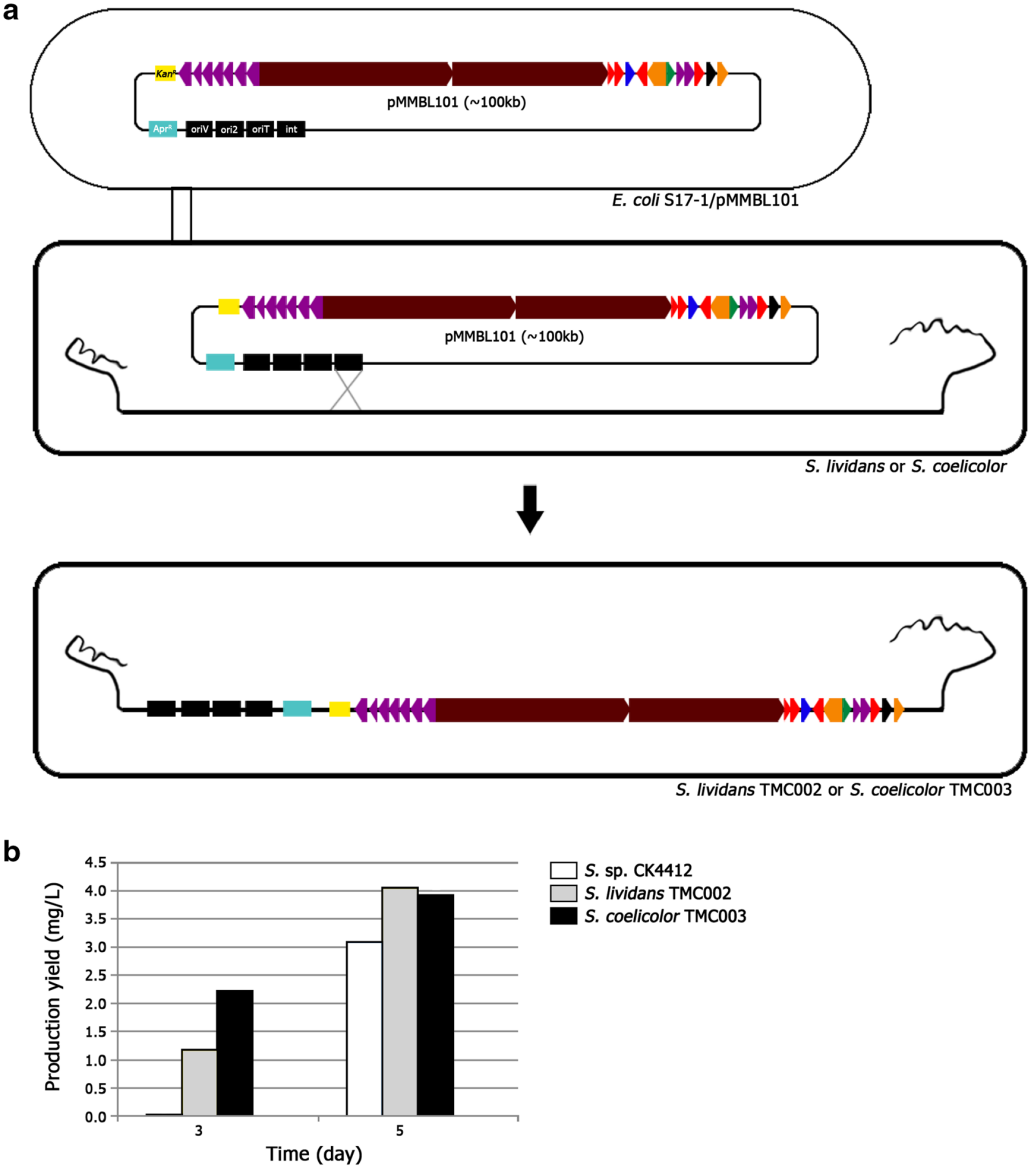
**a** Construction of *S.* *lividans* TMC002 and *S. coelicolor* TMC003 by *E. coli*-*Streptomyces* conjugation system. **b** Comparison of TMC production yields on 3 and 5 day in CK4412, *S. lividans* TMC002 and *S. coelicolor* TMC003. *White* CK4412, *Gray* *S. lividans* TMC002, *Black* *S. coelicolor* TMC003

The newly formed pMMBL101 vector was conjugated into *Streptomyces* strains, including *S. coelicolor* M145 and *S. lividans* TK21. Both *S. lividans* and *S. coelicolor* have been successfully used for the heterologous expression of various *Streptomyces* secondary metabolite biosynthetic gene clusters. pMMBL101 was first transferred into *S. lividans* TK21 via conjugation, and the resulting transformant strain containing the *tmc* gene cluster was named *S. lividans* TMC002 (Fig. 3a). pMMBL101 was also introduced into *S. coelicolor* M145 by PEG-mediated transformation, resulting in *S. coelicolor* TMC003. These two recombinant strains along with wild-type strain were cultured in R5 media for 5 days. Although TMC was not detected in the 3-day wild-type culture, both *S. lividans* TMC002 and *S. coelicolor* TMC003 showed TMC production by day 3 (Fig. 3b). After 5 days of culture, TMC production levels in TMC002 and TMC003 were about 1.3-fold (4.05 mg/L) and 1.26-fold (3.91 mg/L) higher than that in wild-type (3.1 mg/L), respectively (Fig. 3b). These results reveal that the pSBAC-driven heterologous expression of an entire TMC biosynthetic gene cluster resulted in rapid and enhanced TMC production.

### Homologous or heterologous tandem integration of entire TMC cluster

**Fig. 4:**
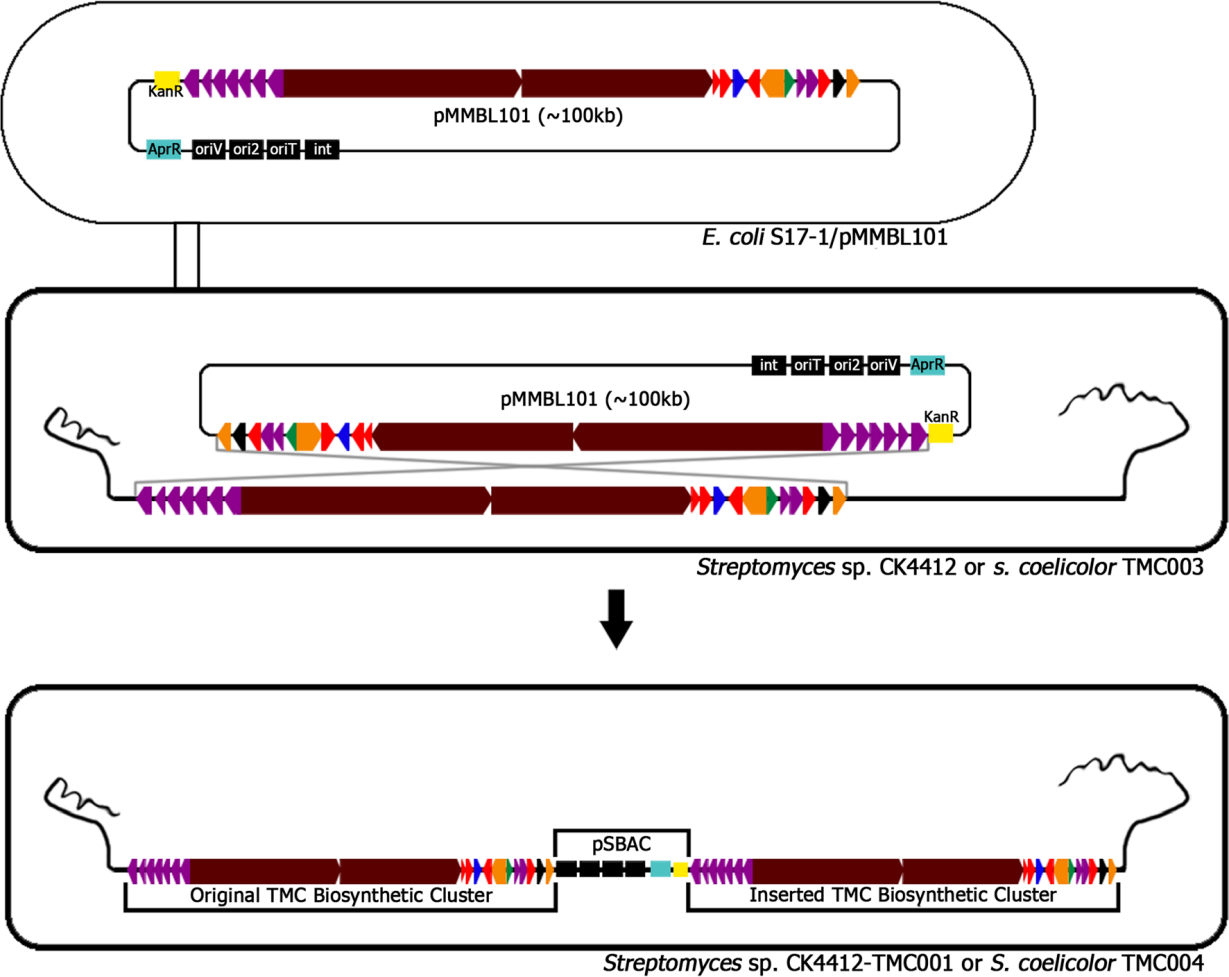
Construction of *Streptomyces* sp. CK4412-TMC001 and *S. coelicolor* TMC004

**Fig. 5:**
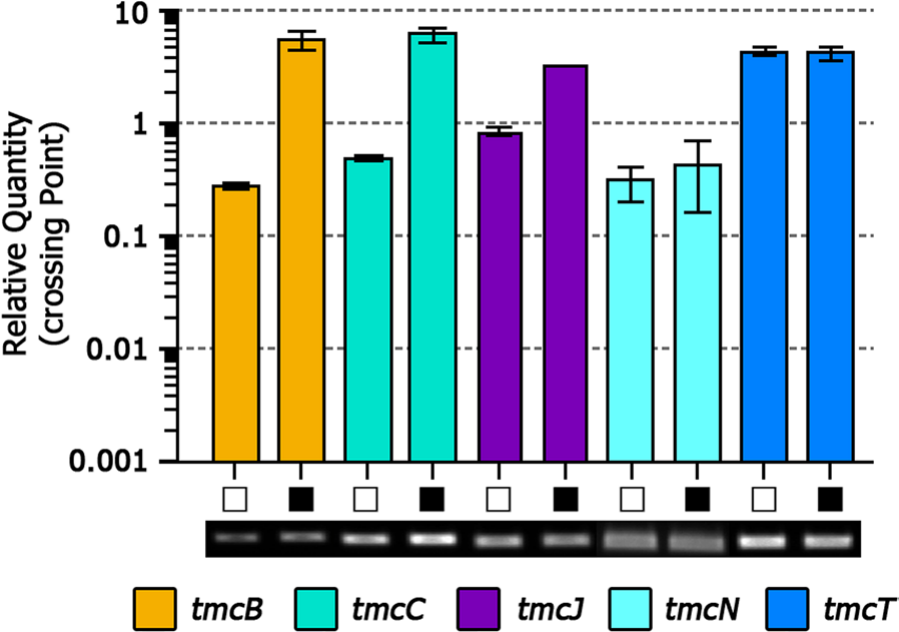
TMC production comparison of heterologous and homologous expression strains

**Fig. 6:**
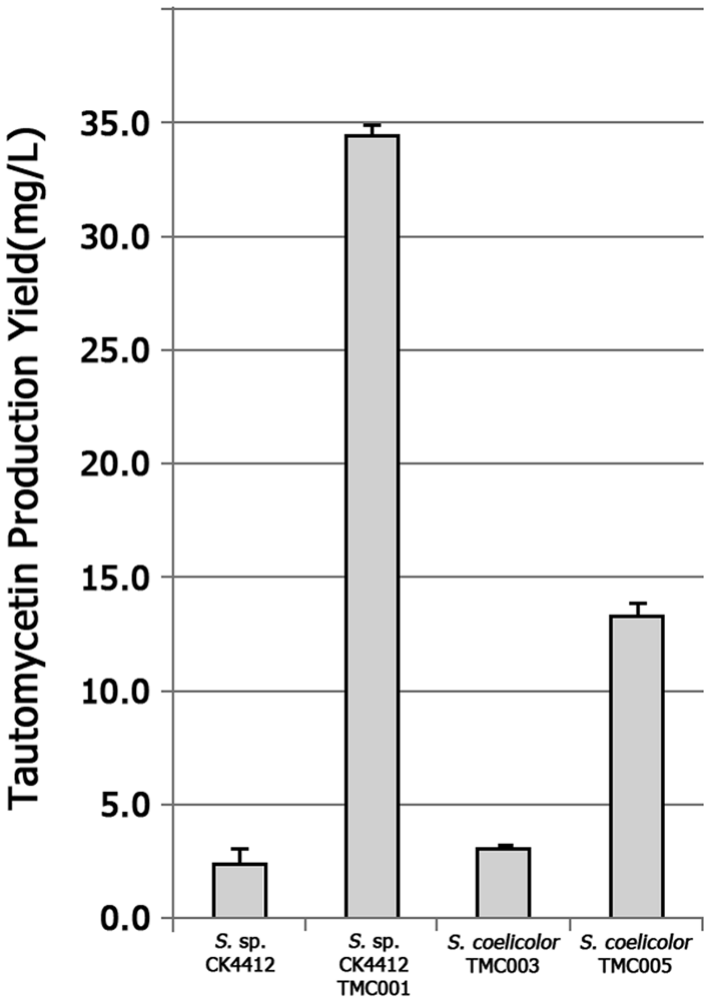
Transcripts analysis of *tmc*-overexpressed CK4412 (CK4412-TMC001). The RNA sample was taken day 5. Three biosynthetic genes (*tmcB, mcC,* and *tmcJ*) and two pathway specific regulatory genes (*tmcN* and *tmcT*) were amplified. Each gene was amplified in duplicate. *Error rates* were calculated by standard deviation. *White square* CK4412 WT; *black square* CK4412-TMC001

To further stimulate TMC productivity, an additional copy of the TMC cluster was introduced into the TMC single copy-containing wild-type *Streptomyces* sp. CK4412 and *S. coelicolor* TMC003 strains (Fig. 4). pMMBL101 was first introduced into *Streptomyces* sp. CK4412 by conjugation. Among the resulting ex-conjugants, four were randomly selected for further analysis by PCR amplification of *attP*-*int*-amplifying primer sets. PCR analysis showed that pMMBL101 integrated adjacent to the original *tmc* cluster in three of the four selected strains (named CK4412-TMC001), whereas pMMBL101 inserted into the *attB* site of the *Streptomyces* sp. CK4412 chromosome in only one strain. Chromosomal integration of pMMBL101 was confirmed by rapid draft genome sequencing. Total length of complete genome sequence was 9,803,578 bp. G + C content was determined to be 71.27 %. From the gene prediction results, 9141 CDSs were identified. The contig arrangements show that the pSBAC was inserted between two TMC biosynthetic gene clusters (Additional file 4: Fig.S3). *Streptomyces* sp. CK4412-TMC001 cultured in R5 media for 5 days showed a 14-fold increase in TMC production (34.47 mg/L) compared to the parental strain (2.47 mg/L) (Fig. 5). Comparative qRT-PCR results also confirmed that transcription of three biosynthetic genes (*tmcB*, *tmcC*, and *tmcJ*) as well as two pathway-specific regulatory genes (*tmcN* and *tmcT*) was significantly stimulated in CK4412-TMC001 (Fig. 6). This implies that the presence of an additional copy of the entire TMC biosynthetic gene cluster was responsible for the increased transcription of TMC biosynthetic genes. To further verify introduction of a tandem repeat of the TMC cluster into a heterologous host, an apramycin-resistant gene of pMMBL101 was replaced by a spectinomycin/streptomycin-resistant gene to generate pMMBL102, followed by introduction into *S. coelicolor* TMC003 (named *S. coelicolor* TMC004). Similar to CK4412-TMC001 containing a tandem repeat of the TMC cluster, *S. coelicolor* TMC004 cultured in R5 media for 5 days also showed a 5.4-fold increase in TMC production (13.31 mg/L) compared to the original TMC-producing strain, *S.* sp. CK4412 (2.47 mg/L) (Fig. 5), suggesting that pSBAC-driven introduction of a cluster tandem repeat was equally effective in both homologous and heterologous host systems.

## Discussion

Isolation and characterization of a secondary metabolite pathway gene cluster in *Streptomyces* species can elucidate its molecular biosynthetic and regulatory mechanisms. Here, a secondary metabolite produced by *Streptomyces* sp. CK4412, originally isolated from Jeju Island, Korea, was identified as an activated T cell-specific immunosuppressive compound with novel pharmacological activities in both in vivo and in vitro studies, and its chemical structure was shown to be identical to that of tautomycetin (TMC), an antifungal compound with a structurally-unique ester bond linkage between its terminal cyclic anhydride moiety and linear polyketide chain [19]. TMC is believed to specifically block tyrosine phosphorylation of intracellular signal mediators downstream of Src tyrosine kinases in a T cell-specific manner via selective inhibition of protein phosphatase 1 (PP1) and PP2A [18, 20, 21]. However, the Src homology-2 domain containing protein tyrosine phosphatase-2 (SHP2) was also recently shown to be a putative target for the immunosuppressive activity of TMC [22]. Moreover, TMC has been reported to inhibit growth of colorectal cancer cells, implying it is a potentially-valuable natural product with multiple medically relevant functions, including anti-fungal, immunosuppressive, and anti-cancer activities. Although the entire TMC biosynthetic and regulatory pathway gene clusters have been isolated from *Streptomyces* sp. CK4412 and characterized, pharmacokinetic application of TMC as a novel bioactive compound remains insufficient due to lack of knowledge of how its multiple biological activities correlate to specific moieties of its structure as well as its intrinsic low-level titer observed in wild-type.

Although heterologous expression of a metabolic pathway gene cluster is an attractive alternative to conventional cell culture, cloning and stable expression of large-sized DNA have become challenges to developing a heterologous expression system for *Streptomyces*. A few recent reports have examined the overexpression of a secondary metabolite gene cluster in *Streptomyces* species. Specifically, a site-specific recombination system was engineered to catalyze tandem amplification of a 35-kb actinorhodin biosynthetic gene cluster in *S. coelicolor* [23]. In this case, the *oriT*-like recombination sites RsA and RsB as well as site-specific relaxase gene *zouA* were inserted into the *S. coelicolor* genome flanking an actinorhodin biosynthetic gene cluster. Recombination between RsA and RsB was followed by *zouA*-dependent DNA amplification, resulting in an average of nine tandem repeats of the actinorhodin gene cluster per genome as well as a 20-fold increase in actinorhodin production [23]. Unfortunately, the actinorhodin cluster in a 110-kb AUD (amplifiable units of DNA) failed to overproduce actinorhodin, implying this approach may not be applicable to overexpression of a large-sized gene cluster in *Streptomyces*. Another study directly cloned a gene cluster via transformation-associated recombination (TAR) in yeast as an alternative approach [24]. Further, a 67-kb cryptic non-ribosomal peptide synthase biosynthetic gene cluster identified by genome mining of the marine actinomycete *Saccharomonospora* sp. CNQ-490 was cloned and expressed in *S. coelicolor*, resulting in production of taromycin A as a new antibiotic related to daptomycin [24]. Although a TAR-based system might be suitable for cloning and expression of a large cryptic gene cluster screened from actinomycetes genome mining, TAR-based cloning must be performed in yeast before intergeneric conjugation into *Streptomyces*. In addition, a tandem repeat of the target gene cluster in a heterologous host is not applicable in a TAR-based expression system.

pSBAC was previously applied for cloning a 95-kb *Streptomyces* meridamycin biosynthetic gene cluster into a heterologous *S. coelicolor* host [12]. Although unique restriction enzyme *Mfe*I sites were absent inside the *mer* cluster, they were present just adjacent to the *mer* cluster in the chromosome. However, most *Streptomyces* secondary metabolite gene clusters, including the *tmc* cluster, have no unique restriction enzyme sites in their border regions, which make the pSBAC system less attractive for heterologous *Streptomyces* expression. To overcome the limited applicability of the pSBAC-based heterologous expression system, we developed a general method for the site-specific introduction of unique restriction enzyme *Xba*I sites into border regions of the *tmc* biosynthetic gene cluster within the *Streptomyces* sp. CK4412 chromosome, as illustrated in Fig. 2. Using this approach, we demonstrated that any restriction enzyme site could be introduced at any specific location of the chromosome. Cloning of a large-sized DNA fragment is challenging due to its in vitro physical instability. An attractive alternative to conventional in vitro cloning is in vivo plasmid rescue. Instead of cloning the entire *tmc* gene cluster into the pSBAC vector in vitro, a small DNA fragment containing the flanking region of the *tmc* gene cluster, *tmcI*, was cloned into pSBAC and underwent in vivo homologous recombination. Simple *Xba*I digestion of total chromosomal DNA followed by self-ligation, *E. coli* transformation, and apramycin selection were performed to recover the giant recombinant pSBAC vector containing the entire *tmc* biosynthetic gene cluster. Finally, *tmc*-containing pSBAC in recombinant *E. coli* was successfully conjugated or transformed into *Streptomyces* species, implying that the described pSBAC approach could be generally applied to heterologous *Streptomyces* expression and synthetic biology. Further, genetic modification of the cluster, including target promoter optimization or specific gene manipulation, could be easily carried out in *E. coli*. Another advantage of the giant recombinant pSBAC system is the ability to re-introduce the entire gene cluster into both the original host chromosome as well as single cluster-containing recombinant host, resulting in a stable tandem repeat of the entire biosynthetic gene cluster. Two copies of the *tmc* gene cluster exhibited a 14-fold increase in TMC production, implying this pSBAC-driven tandem repeat approach is very effective for both homologous and heterologous overexpression [25, 26]. In conclusion, the *Streptomyces* pSBAC-driven precise cloning and tandem integration approach described here can be applied to the versatile overexpression of a large secondary metabolite gene clusters in actinomycetes, including novel cryptic clusters identified by genome mining [27].

## Conclusions

Although actinomycetes continue to be a rich source of valuable secondary metabolites, their wild-type production levels are usually too low to be useful in the field of drug development. Here, we demonstrated an attractive genetic system for the efficient homologous and heterologous overexpression of a target cluster in *Streptomyces* species. Site-specific chromosomal integration of unique restriction sites as well as in vivo plasmid rescue of a *Streptomyces* bacterial artificial chromosome vector, pSBAC, containing a large biosynthetic gene cluster were performed for precise cloning and expression of the target cluster in a surrogate *Streptomyces* host. Re-introduction of a giant recombinant pSBAC vector into the chromosome of the *Streptomyces* host containing a single copy cluster resulted in a chromosomal tandem repeat of the entire TMC cluster with significantly enhanced TMC productivities. This approach demonstrates a platform technology for the precise cloning and functional overexpression of the entire biosynthetic gene cluster of any potentially-valuable low-titer metabolite in actinomycetes.

## Methods

### Bacterial strains and culture media

Various strains and plasmids used in this study are summarized in Table 1. *E. coli* strains were cultured at 37 °C in Luria–Bertani (LB) broth or on Luria–Bertani agar supplemented with appropriate antibiotics [28]. *Streptomyces* sp. CK4412 was used as an original TMC-producing stain [17]. For production of TMC, all *Streptomyces* strains were grown at 28 °C in TSB media for 2 days and then cultured for 7 days in R5 media [17]. Modified ISP4 medium was used for conjugation while R2YE medium was used for PEG-mediated transformation.

**Table 1:** Bacterial strains and plasmids used in this study

Strain/plasmid	Relevant characteristics	Source/reference
Plasmid		
pSBAC	*aacIII*(IV), *oriT*, *attP*-*int*, backbone of pCC1BAC	[12]
pSATNI	Modified pSBAC which deleted *attP*-*int* and inserted *Kan* ^*R*^ and *tmcI* fragment	This work
pTMC	pSATNI with 85 kb DNA insert containing whole *tmc* gene cluster	This work
pMMBL101	pTMC with *attP*-*int*	This work
pMMBL102	pMMBL101 which replaced *Apr* ^*R*^ into *Spe* ^*R*^	This work
*E. coli*		
EPI300	F- *mcrA*-*D*(*mrr*-*hsd*RMS-*mcrBC*) *trfA* host for cloning and amplification of various BAC vectors and constructs derived from it	Epicenter
S17-1	*E. coli* host for transferring various plasmids into *Streptomyces* via conjugation	
ET12567/pUZ8002	*E. coli* host for transferring various plasmids into *Streptomyces* via conjugation	
*Streptomyces* sp.		
CK4412	Original TMC-producing strain	[17]
CK4412-2XB	CK4412 with pSATNI and *Xba*I recognition sequences in both flanking region of TMC biosynthetic gene cluster	This work
TMC001	pMMBL101-containing CK4412	This work
*Streptomyces lividans*		
TK21	Non TMC-producing strain	
TMC002	TK21 with pMMBL101	This work
*Streptomyces coelicolor*		
M145	Non TMC-producing strain	
TMC003	M145 with pMMBL101	This work
TMC004	pMMBL102-containing *S. coelicolor* TMC003	This work

### Insertion of unique *Xba*I recognition sequences in both flanking regions of tautomycetin biosynthetic gene cluster

To isolate the TMC biosynthetic gene cluster, unique *Xba*I recognition sequences were inserted into both flanking regions of the TMC biosynthetic gene cluster using a PCR-targeted gene disruption system [29]. Briefly, an apramycin resistance gene (*aac(3)IV*)/oriT cassette and spectinomycin resistance gene (*aadA*)/oriT cassette were used to insert *Xba*I recognition sequences into both flanking regions. These cassettes were amplified from pIJ773 and pIJ778 using *Xba*I recognition sequence-containing primers and then introduced into *E. coli* BW25113/pIJ790 containing pTMC2982 or pTMC2290, resulting in pTMC2982::*aac(3)IV*/oriT and pTMC2290::*aad*/oriT, respectively. Insertion of *Xba*I recognition sequences was confirmed by PCR applied to mutated pTMC2982 and pTMC2290. The mutated cosmids pTMC2982::*aac(3)IV*/oriT and pTMC2290::*aad*/oriT were then introduced into *Streptomyce* sp. CK4412 by conjugation with *E. coli* ET12567/pUZ8002. Conjugation experiments were performed as described previously [30]. Conjugation was repeated using the isolated *Streptomyces* sp. CK4412::*aac(3)IV* strains in order to insert the *Xba*I recognition sequence into the opposite flanking region. The desired double cross-over mutants, selected by their apramycin-resistant (or spectinomycin-resistant) and kanamycin-sensitive phenotypes, were isolated. Their genotypes were verified using PCR.

### Isolation of entire tautomycetin biosynthetic gene cluster into pSBAC

To isolate the entire TMC biosynthetic gene cluster from the chromosome by *Xba*I digestion and ligation, *attP*-*int* containing *Xba*I recognition sequences was removed from pSBAC by *Avr*II digestion and ligation. To select the right colony, a kanamycin resistance gene was ligated into *Bam*HI-*Eco*RI-digested pSBAC. To integrate the modified pSBAC into the desired location by homologous recombination, a 3480-bp DNA fragment including a part of *tmcI* (*tmcI′*) was amplified by PCR using the pTMC2982 cosmid as a template. The amplified PCR products were then ligated into a RBC T&A cloning vector. The ligated vector was completely sequenced in order to ensure its integrity (Macrogen, Korea). The *tmcI’* fragment, digested using *Bam*HI and *Hind*III, was cloned into modified pSBAC to generate pSATNI. Conjugation was performed to integrate pSATNI into the chromosome by homologous recombination. The desired mutant (named CK4412-2XB) was selected on kanamycin-included MS agar medium, and its genotypes were verified using PCR.

CK4412-2XB strain was cultured at 28 °C in TSB media for 2 days, and preparation of genomic DNA of CK4412-2XB was carried out using a Wizard^®^ genomic DNA purification kit (Promega). Genomic DNA was digested by restriction enzyme *Xba*I, purified, and concentrated by ethanol precipitation before self-ligation using T4 ligase (TaKaRa). After desalting, the ligation mixture was used for electroporation of *E. coli* EPI300. Recombinant colonies were selected onto apramycin- and kanamycin-containing LB medium. Plasmids were isolated by alkali denaturation and screened by PCR using randomly selected primers within the *tmc* cluster to identify pTMC.

A 2-kb DNA fragment containing the *attP*-*int* of ΦBT1 was amplified by PCR using pSBAC as a template and ligated into RBC T&A cloning vector. The ligated vector was completely sequenced to ensure integrity. The *attP*-*int*, digested using *Avr*II, was cloned into pTMC to generate pMMBL101.

### HPLC quantification and antifungal bioassay for TMC

Extraction of TMC and HPLC analysis were carried out according to previously reported methods [30]. Briefly, culture broth was extracted twice using an equal volume of ethyl acetate, followed by concentration using a rotary evaporator. The final extracts were dissolved in methanol. Analytical HPLC was carried out on a Grace C18 4-μm column at a flow rate of 1 ml/min with UV detection at 273 nm.

Antifungal bioassay was performed for qualitative analysis of TMC production yield of *tmc*-containing heterologous hosts. Evaluation of TMC production was carried out by the paper disc diffusion method. *Aspergillus niger* stock (1 ml) was inoculated onto ME agar medium, after which extract-soaked discs were placed onto the prepared medium. The plates were incubated at 30 °C for 2 days.

### Isolation of total RNA and gene expression analysis by RT-PCR

For RNA preparation, CK4412, CK4412-TMC001, *S. lividans* TMC002, *S. coelicolor* TMC003, *S. coelicolor* TMC004 were grown for 5 days in R5 medium, after which samples were taken at 120 h. Mycelia were harvested by centrifugation and stored in a −40 °C deep freezer after washing twice with distilled water. RNA preparation and RT-PCR were carried out according to previously reported methods [25]. Briefly, after the frozen mycelia were broken by shearing in a mortar, total RNA was isolated by using RNeasy mini kit [QIAGEN, Germany]. DNase-I treated RNA, AVM Reverse Transcriptase XL [TaKaRa, Japan] and random hexamers was used for cDNA synthesis. *HrdB* gene was used as internal control. Transcripts from three biosynthetic genes such as *tmcB, tmcC,* and *tmcJ* and two regulatory genes, *tmcN* and *tmcT*, were analyzed after 30 PCR cycles. Primers used for RT-PCR were previously reported [25].

### Replacement of apramycin-resistant gene by spectinomycin-resistant gene and introduction into *tmc*-containing Strains

To introduce the *tmc* cluster into *S. coelicolor* TMC003, the apramycin-resistant gene of pMMBL101 was replaced by a spectinomycin-resistant gene (named pMMBL102) using a Quick & Easy BAC modification kit (GeneBridges). The Red/ET plasmid was introduced into pMMBL101-containing *E. coli* EPI300, after which BAC modification was performed according to the manufacturer’s guide using PCR to amplify the spectinomycin-resistant gene in the aprR-homologous region. Transformants were selected on spectinomycin-containing LB medium and confirmed by PCR. After pMMBL102 was transformed into *E. coli* S17-1, it was introduced into *S. coelicolor* TMC003 by conjugation.

### Rapid genome sequencing of *Streptomyces* sp. CK4412-TMC001

Genomic DNA of *Streptomyces* sp. CK4412-TMC001 was prepared from 3-days culture with a Wizard^®^ Genome DNA Purification Kit (Promega). The genomic DNA was fragmented by dsDNA fragmentase to make proper size for library construction. Resulting DNA fragments was processed to Illumine Nextera DNA sample preparation kit (Illumina, Inc., USA) following manufacturer’s instruction. Final library was quantified by Bioanalyzer 2100 (Agilent, USA) and average library size was 300 bp.

The genomic library was sequenced by Illumina MiSeq (Illumina, Inc., USA). Generated paired-end sequencing reads (23,891,700 method reads) were assembled using CLC genomics workbench 6.0 (CLC bio, Denmark) and resulted in 253 contigs. The contigs and PCR-based long reads were combined through manual curation by using CodonCode Aligner 3.7.1 (CodonCode Corp., Dedham, MA, USA). The final plasmid sequence was corrected by remapping with raw reads to check errors and dubious regions.

The coding sequences (CDS) were predicted by Glimmer 3.02 [31]. tRNA were identified by tRNA-Scan-SE [32], and rRNA were searched using HMMER with EzTaxon-e rRNA profiles [33, 34]. The predicted CDSs were compared to catalytic families (catFam) and NCBI COG by rpsBLAST and NCBI reference sequences (RefSeq) and SEED databases by BLASTP for functional annotation [35–38].

## Additional files

Additional file 1:
**Table S1.** Primers used in this study. Additional file 2:
**Figure S1.** Confirmation of *Xba*I insertion in both flanking region of TMC biosynthetic gene cluster (A) Diagram of *Xba*I inserted both flanking region of *tmc* cluster (B) PCR analysis of constructed strain. Left, confirmation of *Xba*I insertion in head of *tmc* cluster; Right, confirmation of *Xba*I instertion in tail of *tmc* cluster; 1 and 2, PCR products from CK4412 tDNA; 3 and 4, PCR products from *Xba*I-inserted CK4412 tDNA; 2 and 4, *Xba*I-digested PCR products. Additional file 3:
**Figure S2.** Confirmation of pMMBL101 (A) PCR analysis using randomly selected *tmc* gene primers (B) Enzyme mapping using various restriction enzyme. C, uncut pMMBL101; 1, *Eco*RI; 2, *Eco*RV; 3, *Nde*I; 4, *Hind*III; 5, *Xba*I-digested pMMBL101; M, λ-*Hind*III DNA ladder. Additional file 4:
**Figure S3.** Sequenced contig organization compared with predicted tandem repeated CK4412-TMC001.
