# Supplementary material for: Precise cloning and tandem integration of large polyketide biosynthetic gene cluster using Streptomyces artificial chromosome system
Source: Microb Cell Fact. 2015 Sep 16;14:140. doi: 10.1186/s12934-015-0325-2 (PMC4573296; doi:10.1186/s12934-015-0325-2)
Supplement: Additional file 1: — Table S1. Primers used in this study. [file 12934_2015_325_MOESM1_ESM.docx]

**Table S1**. Used primers in this study

| Primers | Sequence (5’-3’) | Description |
| --- | --- | --- |
| INApr-oriT_F | CTCCCAATCACCGGGCCGATGAGACAGCCGTACCCGGTCATTCCGGGGATCCGTCGACC | *AprR-oriT* insertion primers |
| INApr-oriT_R | GAAGCAGCTCCAGCCTACATCTAGACCCGGACGAGGAGCAAGTGGCCCTCGCCGCGCT |  |
| INSpe-oriT_F | GCATGTCTGCTGCGTGTCGGCAGCAAGCAACGGTCTAGAATTCCGGGGATCCGTCGACC | *SpeR-oriT* insertion primers |
| INSpe-oriT_R | GAAGCAGCTCCAGCCTACAAAGCCGTACGAGCTGCTGCGCCAGGCGTTGGAGCGGTCC |  |
| CHxbaF_F | ATCCACAGCATCGCGTGA | Check primers for insertion of *Xba*I in front of TMC biosynthetic cluster |
| CHxbaF_R | TGCAGCGGGACACGTACA |  |
| CHxbaB_F | CATGAACCTGCAGAGTTC | Check primers for insertion of *Xba*I in back of TMC biosynthetic cluster |
| CHxbaB_R | TCCACGCGAACTTCGTCA |  |
| CLtmcI_F | GGATCCTGGCCGCAGATCGAGATGACGAGCGTCA | Primers for cloning of *tmcI* |
| CLtmcI_R | AAGCTTGCTATGTACTGCTGATCGTCACACTGAC |  |
| CLkanR_F | GAATTCCTCTAGCTAGACCAGAATCG | Primers for cloning of *KanR* |
| CLkanR_R | GGATCCCATGCCTGCAGGTCGACCT |  |
| CLattP_F | CCTAGGATGTCGCCCTTCATCGC | Primers for cloning of *attP-int* |
| CLattP_R | CCTAGGCTACAGCGCCGCAAGCT |  |
| attP-seq_F | ATGGTTATGGCAGCACTGCAT | *AttP-int* check primers for tandem repeated strains |
| attP-seq_R | TATTATCCCGTATTGACGCCG |  |
