# Supplementary figures and images for: Precise cloning and tandem integration of large polyketide biosynthetic gene cluster using Streptomyces artificial chromosome system
Source: Microb Cell Fact. 2015 Sep 16;14:140. doi: 10.1186/s12934-015-0325-2 (PMC4573296; doi:10.1186/s12934-015-0325-2)

**Supplementary Figure 1**

**
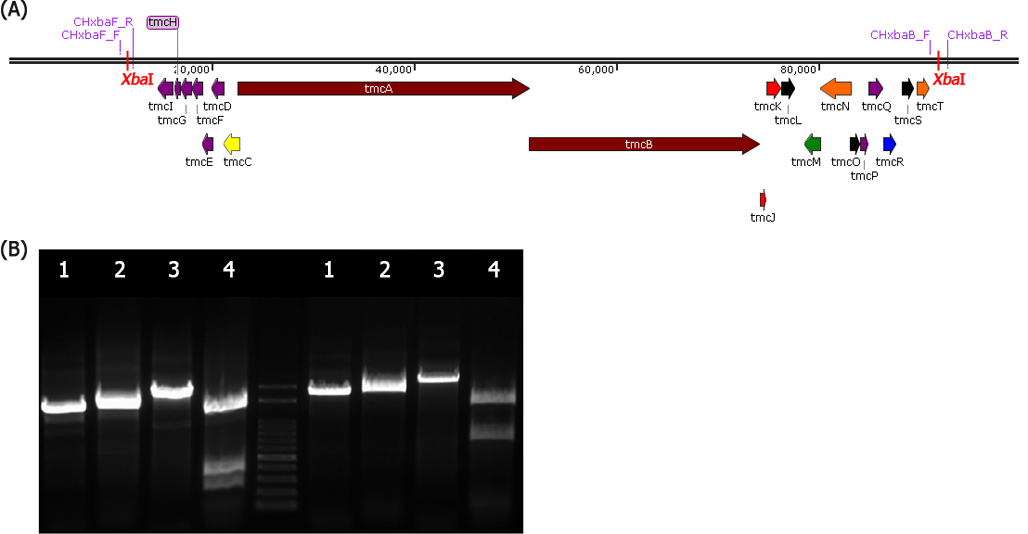
**

Supplement: Additional file 2: — Figure S1. Confirmation of XbaI insertion in both flanking region of TMC biosynthetic gene cluster (A) Diagram of XbaI inserted both flanking region of tmc cluster (B) PCR analysis of constructed strain. Left, confirmation of XbaI insertion in head of tmc cluster; Right, confirmation of XbaI instertion in tail of tmc cluster; 1 and 2, PCR products from CK4412 tDNA; 3 and 4, PCR products from XbaI-inserted CK4412 tDNA; 2 and 4, XbaI-digested PCR products. [file 12934_2015_325_MOESM2_ESM.docx]

**Supplementary Figure 2**

**
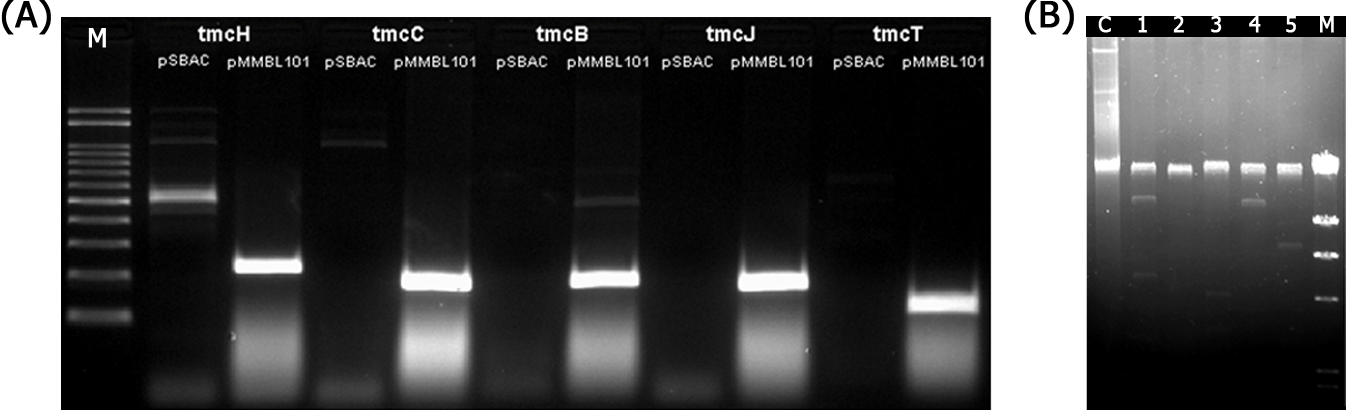
**

Supplement: Additional file 3: — Figure S2. Confirmation of pMMBL101 (A) PCR analysis using randomly selected tmc gene primers (B) Enzyme mapping using various restriction enzyme. C, uncut pMMBL101; 1, EcoRI; 2, EcoRV; 3, NdeI; 4, HindIII; 5, XbaI-digested pMMBL101; M, λ-HindIII DNA ladder. [file 12934_2015_325_MOESM3_ESM.docx]

**Supplementary Figure 3**

**
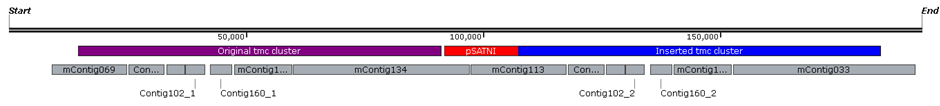
**

Supplement: Additional file 4: — Figure S3. Sequenced contig organization compared with predicted tandem repeated CK4412-TMC001. [file 12934_2015_325_MOESM4_ESM.docx]
